# SnowPole Detection: A comprehensive dataset for detection and localization using LiDAR imaging in Nordic winter conditions

**DOI:** 10.1016/j.dib.2025.111403

**Published:** 2025-02-20

**Authors:** Durga Prasad Bavirisetti, Gabriel Hanssen Kiss, Petter Arnesen, Hanne Seter, Shaira Tabassum, Frank Lindseth

**Affiliations:** a Department of Computer Science, Norwegian University of Science and Technology, Trondheim, Norway; b Department of Mobility, SINTEF AS, Trondheim, Norway; c Department of Computer Science, University of Gävle, Gävle, Sweden

**Keywords:** Snow pole detection, LiDAR images, Dataset, Computer vision, Autonomous vehicles, Geospatial localization

## Abstract

The **SnowPole Detection** dataset is a comprehensive collection of labeled LiDAR images, specifically designed for snow pole detection in road environments. This dataset was collected using a high-resolution **OS2-128 LiDAR** sensor mounted on an autonomous vehicle research platform, covering diverse environments such as mountainous, open, and forested areas. The SnowPole Detection dataset supports applications in computer vision, with a particular focus on *snow pole detection* and *localization*. he OS2-128 LiDAR sensor captures point clouds, which are processed using the Ouster SDK to generate 360-degree images in four modalities: Near-IR, Signal, Reflectivity, and Range. To enhance usability, color images were generated by assigning the first three modalities (Near-IR, Signal, and Reflectivity) to the blue, green, and red channels, respectively, excluding the Range modality. Initial labeling was conducted using **Roboflow**, with further refinement in **CVAT**, resulting in high-quality annotations. The dataset comprises a total of 1,954 manually labeled images, divided into 1,367 training images, 390 validation images, and 197 test images, following a 70/20/10 split. Since the images across all modalities are pixel-aligned, the labels for the color images are also applicable to each modality individually. This structure allows researchers to directly use the dataset for snow pole detection tasks, whether focusing on color or individual LiDAR modalities. The SnowPole Detection dataset is publicly available at Mendeley^1^.

Specifications Table

The Specifications Table provides a concise overview of the SnowPole Detection dataset, including details on data type, acquisition methods, and the collection process across diverse terrains in Norway.

**Table:** 

Subject	Object Detection, Computer Vision, Geospatial Information Science, Deep Learning, Artificial Intelligence
Specific Subject Area	Snow Pole Detection and Localization using LiDAR Imaging
Type of Data	360-degree Images (.png), Annotation Files (.txt)
Data Acquisition Method	The OS2-128 LiDAR sensor mounted on an instrumented vehicle (see Fig. 1) captured 360-degree images across four modalities: Near-IR, Signal, Reflectivity, and Range.
Data Format and Size	Labeled images in.png format with YOLO-format labels [1], Image Width: 1024 pixels, Height: 128 pixels
Experimental Factors	Data captured in varied terrains, including mountainous regions, forests, and rural roads
Experimental Features	High-resolution LiDAR images annotated with snow pole labels
Data Source Location	E39 Hemnekjølen, Norway (see Fig. 5)
Description of Data Collection	Color images were generated by assigning Near-IR, Signal, and Reflectivity modalities to the blue, green, and red channels, respectively (excluding Range). Initial labeling was performed using Roboflow, followed by refinement in Computer Vision Annotation Tool (CVAT) [2] for high-quality annotations.
Data Accessibility	Publicly available on Mendeley Data at SnowPole Detection (Data identification number: 10.17632/tt6rbx7s3h.2).

Specifications table for the SnowPole Detection dataset	

## 1 Value of the Data

The SnowPole Detection dataset [3] offers a specialized resource for the automatic detection of snow poles in Nordic environments. This dataset is valuable for infrastructure management and autonomous systems in challenging climates and expands upon our research on snow pole detection and geospatial localization using a Light Detection and Ranging (LiDAR) - Global Navigation Satellite System (GNSS) data fusion framework [4].

• **Support for Model Development**: The SnowPole Detection dataset enables the development and testing of deep learning models optimized for Nordic conditions. Researchers may leverage this dataset to train and validate algorithms that need to perform in snowy and icy environments.

**Fig. 1:**
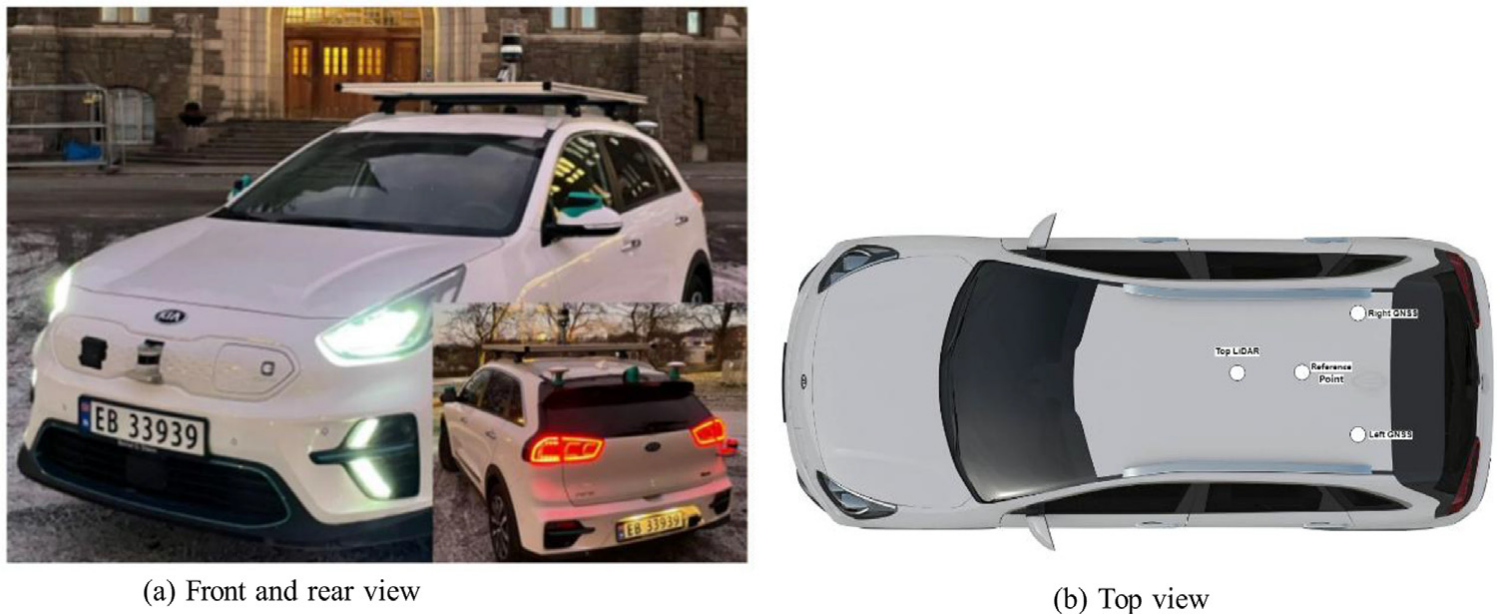
The NAPLab Research Platform used for data collection.
• **Adaptability to Nordic Climates**: As data is captured entirely in Norway, the dataset mirrors the challenges specific to Nordic climates, making it particularly relevant for infrastructure projects and autonomous systems in similarly harsh conditions.
• **Focused Snow Pole Detection**: The dataset’s emphasis on precise snow pole detection supports winter safety assessments, traffic infrastructure projects, and environmental monitoring, providing a foundation for expanded applications to similar objects in future research.
• **Benchmarking and Performance Assessment**: SnowPole Detection dataset serves as a valuable benchmarking tool for machine learning researchers. It allows for algorithm evaluation in complex winter conditions, offering insights essential for real-world applications in challenging climates.
• **Suitability for Competitions and Collaborative Challenges**: This dataset is well-suited for competitions addressing object detection in winter environments, fostering collaboration and innovation in managing adverse weather conditions.

## 2 Data Description

**Fig. 2:**
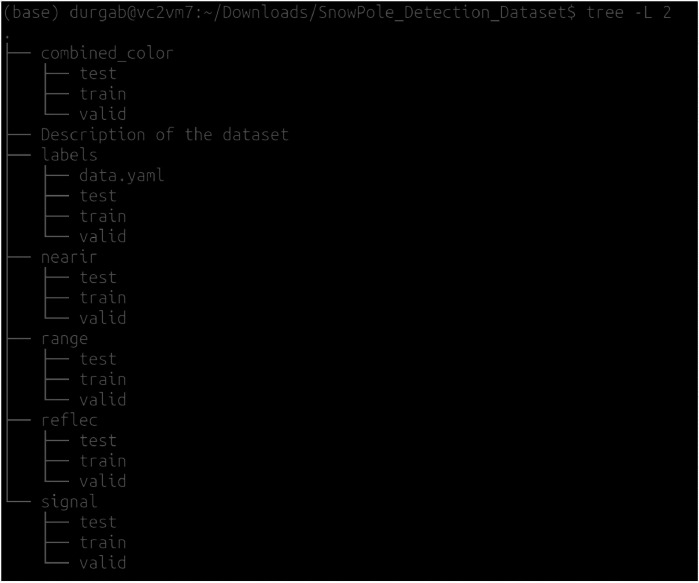
Structure of the SnowPole Detection dataset for LiDAR-based snow pole detection and localization.

**Fig. 3:**
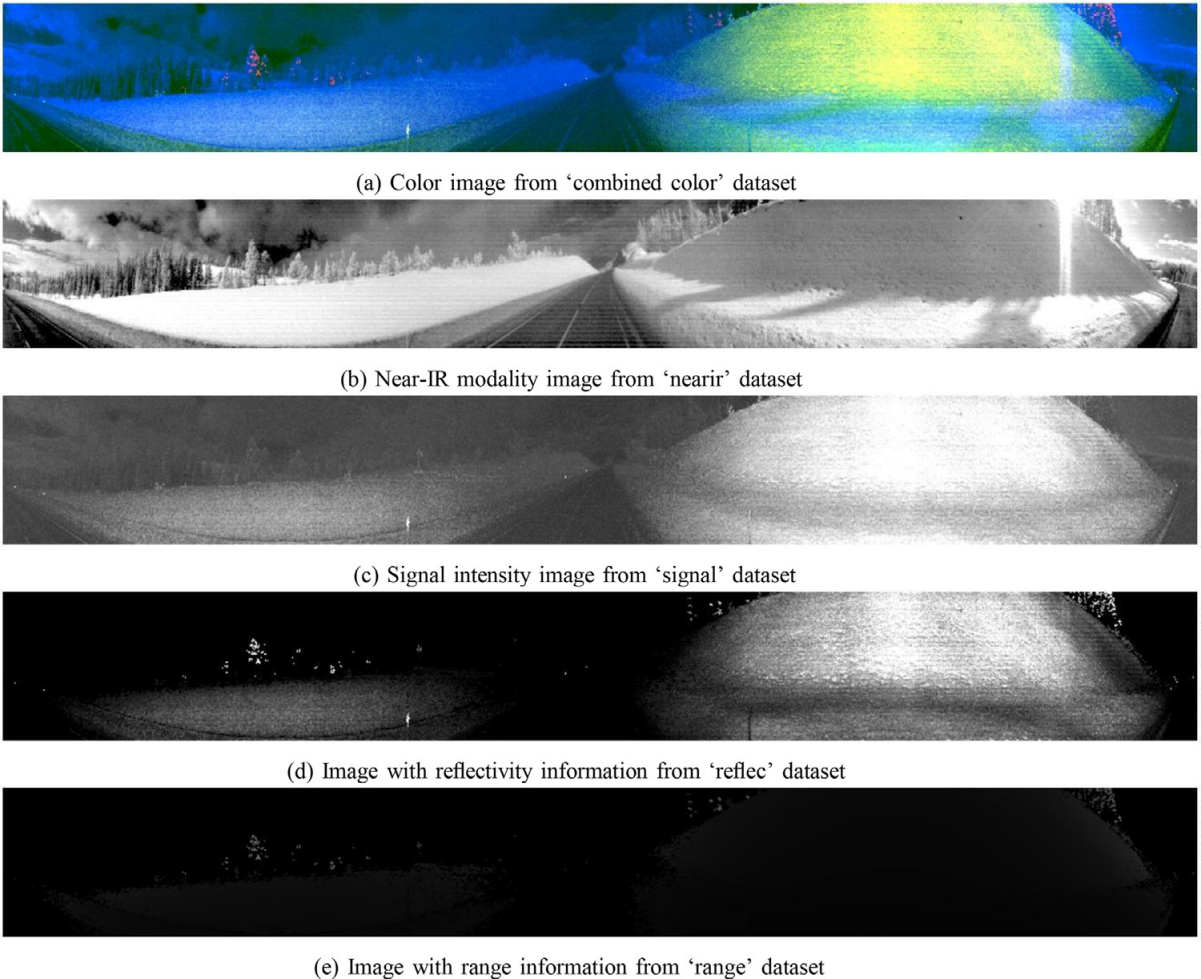
Visualization of image_1967.png from the SnowPole Detection Dataset

The SnowPole_Detection_Dataset on Mendeley is organized into multiple folders, each representing a different LiDAR modality or data component. The dataset is split into training (train), validation (valid), and testing (test) sets, following a 70/20/10 split. Each modality folder contains images, with annotations provided in YOLO format, applicable across all modalities. Fig. 2 illustrates the structure of the SnowPole Detection dataset, while Fig. 3 shows sample images from each modality folder.

• **combined_color/**: Contains color images created by mapping three LiDAR modalities to BGR channels (Near-IR, Signal, and Reflectivity mapped to blue, green, and red channels, respectively).
  – **train/**: Training images in combined color format.
  – **valid/**: Validation images in combined color format.
  – **test/**: Test images in combined color format for final model evaluation.
• **labels/**: YOLO-format annotations specifying snow pole locations and classes, applicable across all image modalities.
  – **train/**: Training labels.
  – **valid/**: Validation labels.
  – **test/**: Test labels for final evaluation.
• **nearir/, signal/, reflec/, range/**: Each folder contains images from a specific LiDAR modality:
  – **nearir/**: Near-infrared images.
  – **signal/**: Signal intensity images.
  – **reflec/**: Reflectivity images.
  – **range/**: Range (distance) images.
  For each modality:
  – **train/**: Training images.
  – **valid/**: Validation images.
  – **test/**: Test images for final model evaluation.

### 2.1 YOLO Label Format for SnowPole Detection Dataset

In the SnowPole Detection dataset, YOLO-format annotations specify the location and class of each detected snow pole within an image. Each label file corresponds to an image and contains several lines, with each line representing one detected snow pole. The format for each line is as follows:

<class_id> <x_center> <y_center> <width> <height>

• **class_id**: An integer representing the class of the object. Since this dataset focuses exclusively on snow pole detection, it has a single class ID (e.g., 0 for poles).
• **x_center** and **y_center**: The normalized coordinates (ranging from 0 to 1) of the pole’s center within the image. x_center is the horizontal position, and y_center is the vertical position. Normalization is done by dividing the center coordinates by the image width and height, respectively.
• **width** and **height**: The normalized width and height of the bounding box around the snow pole, also ranging from 0 to 1. These values represent the dimensions of the box containing the snow pole, relative to the image dimensions.

#### 2.1.1 Example Label Entry

For an image in the SnowPole Detection dataset, a label file might contain lines such as:

0 0.524 0.398 0.082 0.200

This line indicates:

• **Class ID**0, representing a snow pole.
• **Center of the bounding box** at 52.4% of the image width from the left and 39.8% of the height from the top.
• **Bounding box width** at 8.2% of the image width and **height** at 20% of the image height.

#### 2.1.2 Applicability to All Modalities

These YOLO annotations are consistent across all image modalities in the SnowPole Detection dataset. Each label file can be applied to images from different channels (e.g., Near-IR, Signal, Reflectivity), allowing uniform snow pole detection and localization across modalities. This design facilitates training and testing across various sensor inputs, ensuring that the model learns pole locations consistently across the different modalities in the dataset.

### 2.2 Visualization of Images from Different Modalities in the SnowPole Detection Dataset

The following Python code (Listing 1) visualizes a specified image across multiple modalities in the SnowPole Detection dataset. Each modality’s image displays the bounding boxes, if available, providing a visual comparison of how the snow pole appears in different sensor outputs.

**Listing 1:**
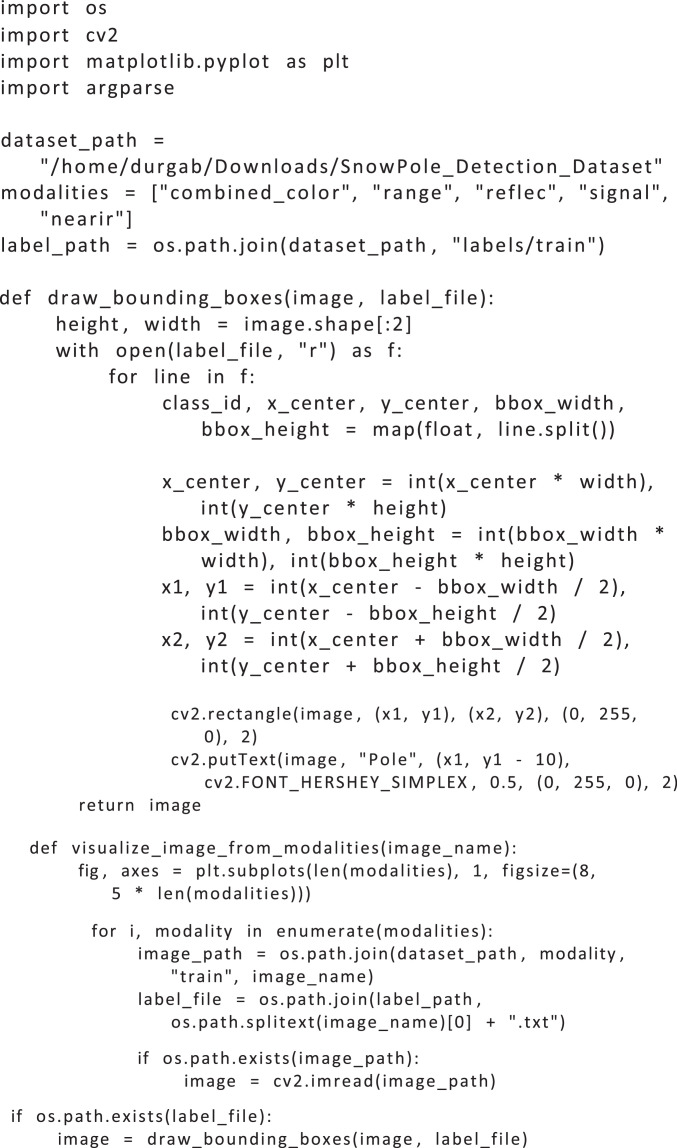

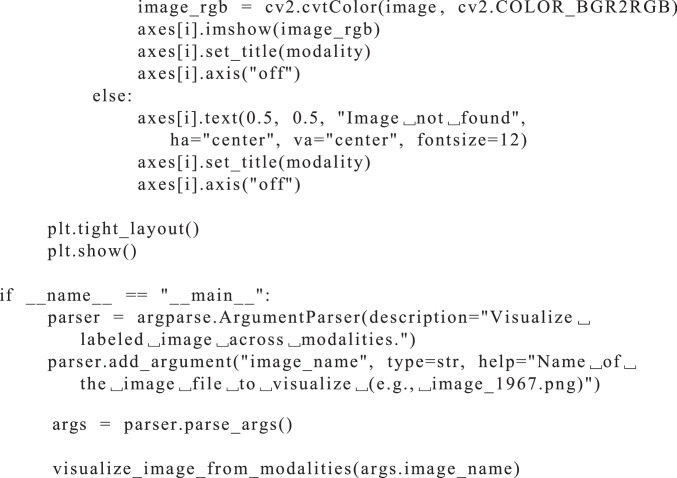
Python Code for Visualizing Image Across Modalities.

As seen in Listing 1, the code provides a detailed method to visualize images across modalities.

#### 2.2.1 Example Usage

To run the code, save it as a Python file (e.g., visualize_modalities.py) and execute it from the command line by specifying an image filename as an argument. For instance:

python visualize_modalities.py image_1967.png

**Fig. 4:**
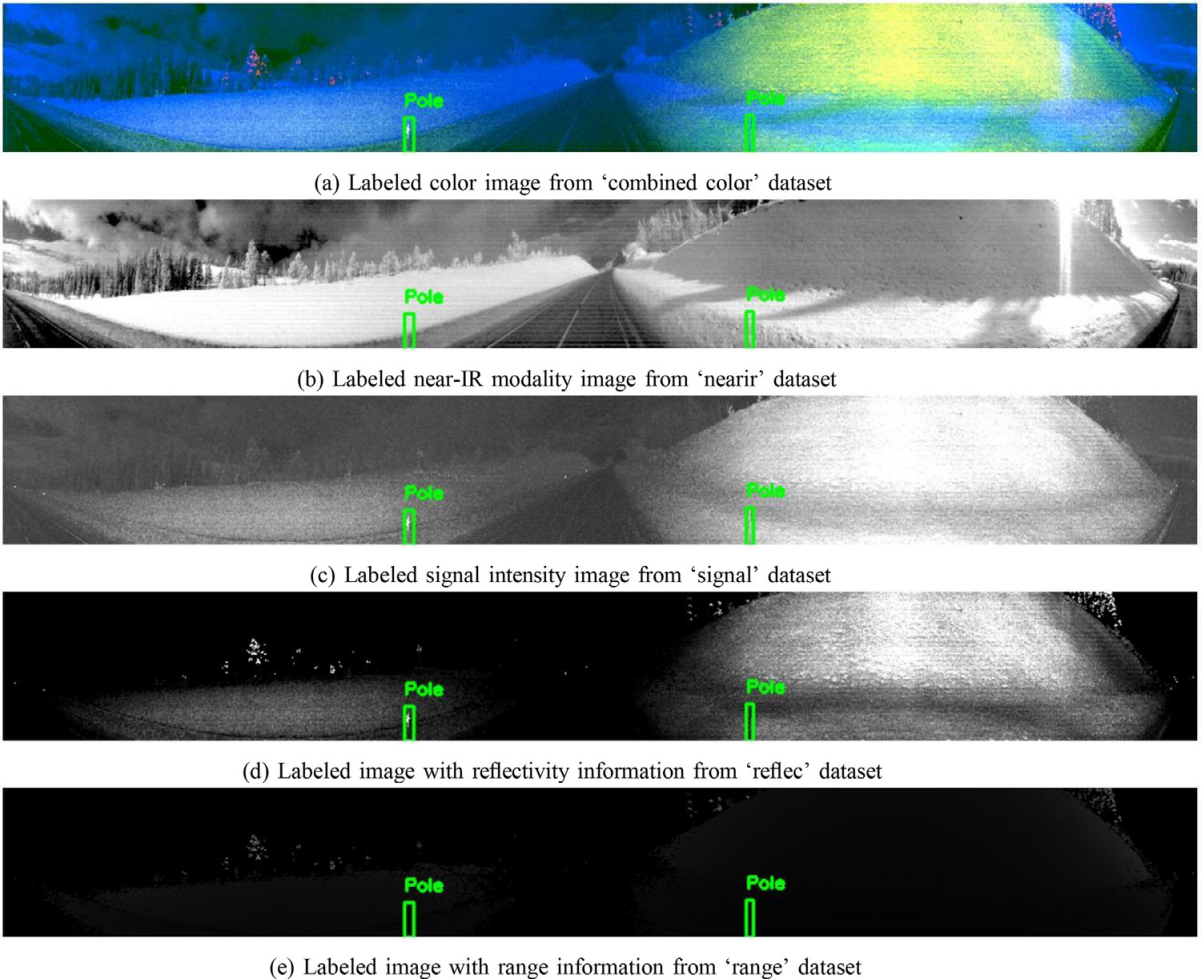
Visualization of image_1967.png with labels from the SnowPole Detection Dataset

This command will generate a vertically arranged display of image_1967.png across all modalities. Each image will have bounding boxes drawn around snow poles (if they are annotated in the label file), providing a clear visual comparison of how the snow pole appears in each sensor modality as shown in Fig. 4.

#### 2.2.2 Code Explanation

The code is structured to accept an image filename as a command-line argument and then visualize the corresponding images from each modality. It consists of several components, explained below:

• **Library Imports**: The code uses the os library for file operations, cv2 (OpenCV) for image processing, matplotlib.pyplot for image display, and argparse to handle command-line arguments.
• **Dataset Paths and Modality List**: Paths are set for the dataset and labels based on the YAML configuration. A list of modalities is defined to facilitate iteration over each modality.
• **Bounding Box Drawing Function**: The function draw_bounding_boxes accepts an image and a corresponding label file, reading the label file in YOLO format (i.e., <class_id> <x_center> <y_center> <width> <height>). Each line in the label file represents one bounding box. The function calculates pixel coordinates from the normalized YOLO coordinates and draws the bounding boxes on the image using OpenCV functions.
• **Visualization Function**: The main function, visualize_image_from_modalities, accepts an image filename as input. It loads the image from each modality’s folder, draws bounding boxes if a corresponding label file exists, and arranges the images vertically for easy comparison. If an image or label file is missing, it displays an error message for that modality.
• **Main Block for Command-Line Arguments**: The if __name__ == ”__main__” block initializes the argument parser, allowing the user to specify an image filename from the command line. The code then calls the visualization function with this filename.

## 3 Experimental Design, Materials, and Methods

**Fig. 5:**
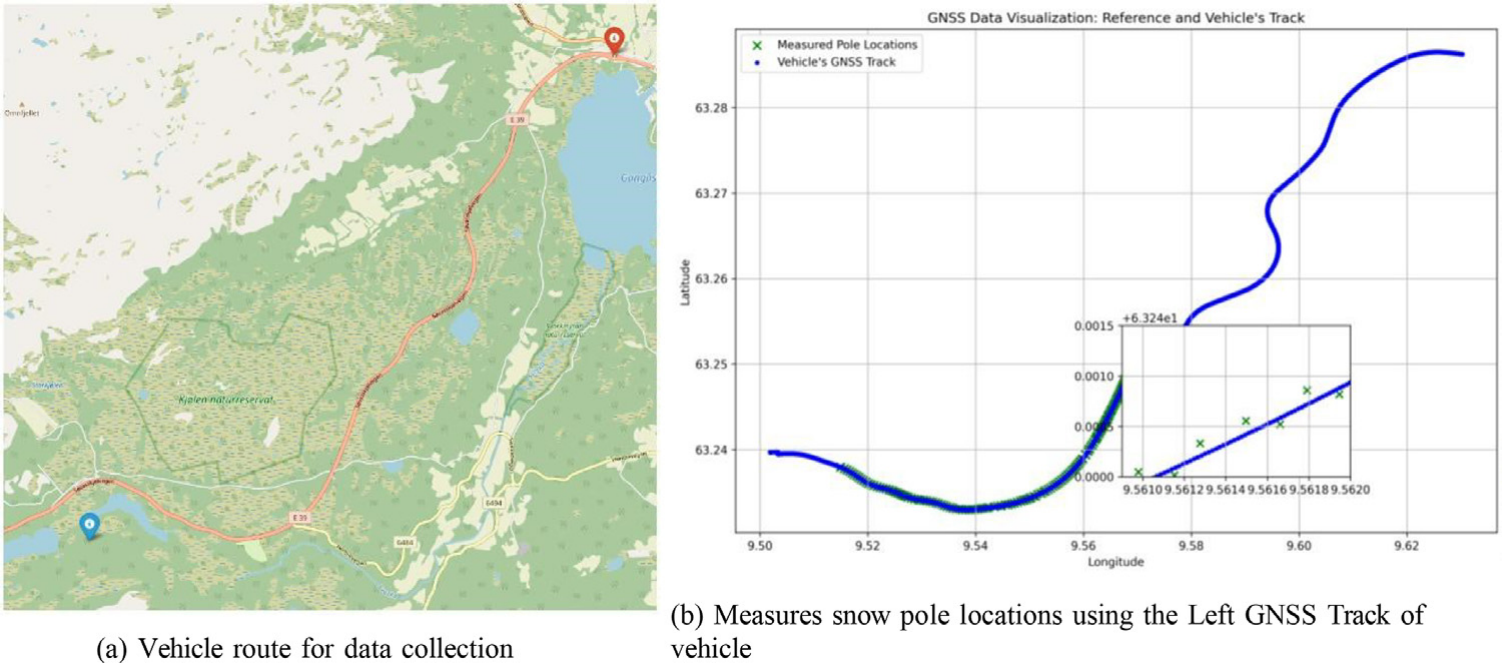
Experimental design at E39 Hemnekjølen.

Data were collected along a 4.2 km route at E39 Hemnekjølen, Norway (as displayed in Fig. 5). The test location features diverse terrain, including mountainous areas, open landscapes, and forested segments. The vehicle, a fully electric Kia e-Niro from NTNU’s NAPLab (as depicted in Fig. 1), was equipped with a 128-channel OS2-128 LiDAR and GNSS system. The figure illustrates the vehicle setup, detailing sensor placements on the vehicle.

Each LiDAR frame was processed using the Ouster Python SDK [5] to generate four image modalities: Near-IR, Signal, Reflectivity, and Range. To enhance usability, a color composite was created by mapping Near-IR, Signal, and Reflectivity to the blue, green, and red channels, respectively, omitting the Range modality.

Snow pole detection is approached as an object detection task. Snow poles were manually annotated through a two-stage labeling process: initial annotations were created in Roboflow [6] and then refined in CVAT [2] to ensure high-quality labeling. This refinement was necessary to mitigate any potential impact of Roboflow’s JPEG compression on the performance of object detection models [7]. Most labeled objects are close to the vehicle, ensuring that only poles are annotated. The dataset comprises 1,954 annotated images, split into 1,367 training images, 390 validation images, and 197 test images, following a 70/20/10 split. Since all modalities are pixel-aligned, annotations can be consistently applied across each modality, offering flexibility for model training. This alignment enables researchers to leverage the training and validation sets to develop models using any object detection framework.

## Data Availability

Mendeley DataSnowPole Detection: A Comprehensive Dataset for Detection and Localization Using LiDAR Imaging in Nordic Winter Conditions (Original data)
